# Head and neck cancer: Emerging concepts in biomarker discovery and opportunities for clinical translation

**DOI:** 10.1002/ctm2.209

**Published:** 2020-11-16

**Authors:** Luiz Paulo Kowalski, Ricardo Della Coletta, Tuula Salo, Mariana Maschietto, Rubens Chojniak, Jefferson Muniz Lima, Alex Mlynarek, Michael P Hier, Moulay A. Alaoui‐Jamali, Sabrina Daniela Silva

**Affiliations:** ^1^ Department of Head and Neck Surgery and Otorhinolaryngology A.C. Camargo Cancer Center and National Institute of Science and Technology on Oncogenomics (INCITO) Sao Paulo Brazil; ^2^ Department of Oral Diagnosis School of Dentistry University of Campinas Sao Paulo Brazil; ^3^ Cancer and Translational Medicine Research Unit University of Oulu Oulu Finland; ^4^ Department of Oral and Maxillofacial Diseases University of Helsinki Helsinki Finland; ^5^ Center of Boldrini Children's Hospital Campinas Brazil; ^6^ Otolaryngology‐Head and Neck Surgery Department Jewish General Hospital–Segal Cancer Center McGill University Montreal Canada; ^7^ Segal Cancer Centre Lady Davis Institute for Medical Research McGill University Montreal Canada

Dear Editor:

In this letter, we update the significant progress made in fundamental research to identify new paradigms in head and neck cancer (HNC), including emerging issues in translating relevant findings into patient‐oriented clinical applications. This overview stems from relevant presentations by renowned leaders in this field given at the Multidisciplinary International Symposium in Translational Head and Neck Cancer Research.

It is noticeable that new cases of HNC are increasing rapidly in the past decades, especially among young adults. This is not only associated with tobacco and alcohol consumption, but due to the HPV infection that has emerged as an additional risk factor, defining a new subtype of tumor that is distinct from the “conventional” HPV‐negative ones.^1^ Like other malignancies, HNC is remarkably heterogeneous comprising several subtypes in different anatomic sites of the upper aerodigestive tracts with complex pathological and molecular features.^1^ However, HNC patients still receive standard treatment based on surgery, radiotherapy, chemotherapy, immunotherapy, or combinations of these modalities, independently of the molecular heterogeneity and/or HPV status. Despite of the advances in medical imaging and therapeutic approaches, the outcome of patients with advanced HNC remains poor and the 5‐years survival is stagnant at <50%.^2^ Indeed, the lack of flexibility in therapeutic strategies often leads to patients suffering from inadequate or excessive treatment. The postoperative follow‐up mode of watchful waiting has also deprived most patients with recurrent HNC of early treatment opportunities.

With no doubt, the identification of reliable and unequivocal diagnostic and prognostic biomarkers is the ultimate goal of clinician and oncoscientists. Basic research in cancer biology provides preclinical data and new technology to support clinical advances. The strategic plan to promote the translational research follows a framework involving different phases (Figure 1).^3^ In order to generate meaningful biological knowledge, an increasing number of investigators are conducting multi‐omics experiments, which allow a rapid and comprehensive analysis of cancer individual patients even using a small biopsy or blood circulating tumor cells.^4^ The ever‐growing amount and sophistication combined with the quantity of data and technical expertise required for data collection and powerful bioinformatics analysis and tools are far from trivial. Nevertheless, the clinical integration of these high‐throughput technologies to offer precision health assessments with valuable therapeutic approaches remains a challenging task for routine implementation on a population scale, which certainly will require further improvements in data acquisition and analysis at reasonable cost effectiveness.

**FIGURE 1:**
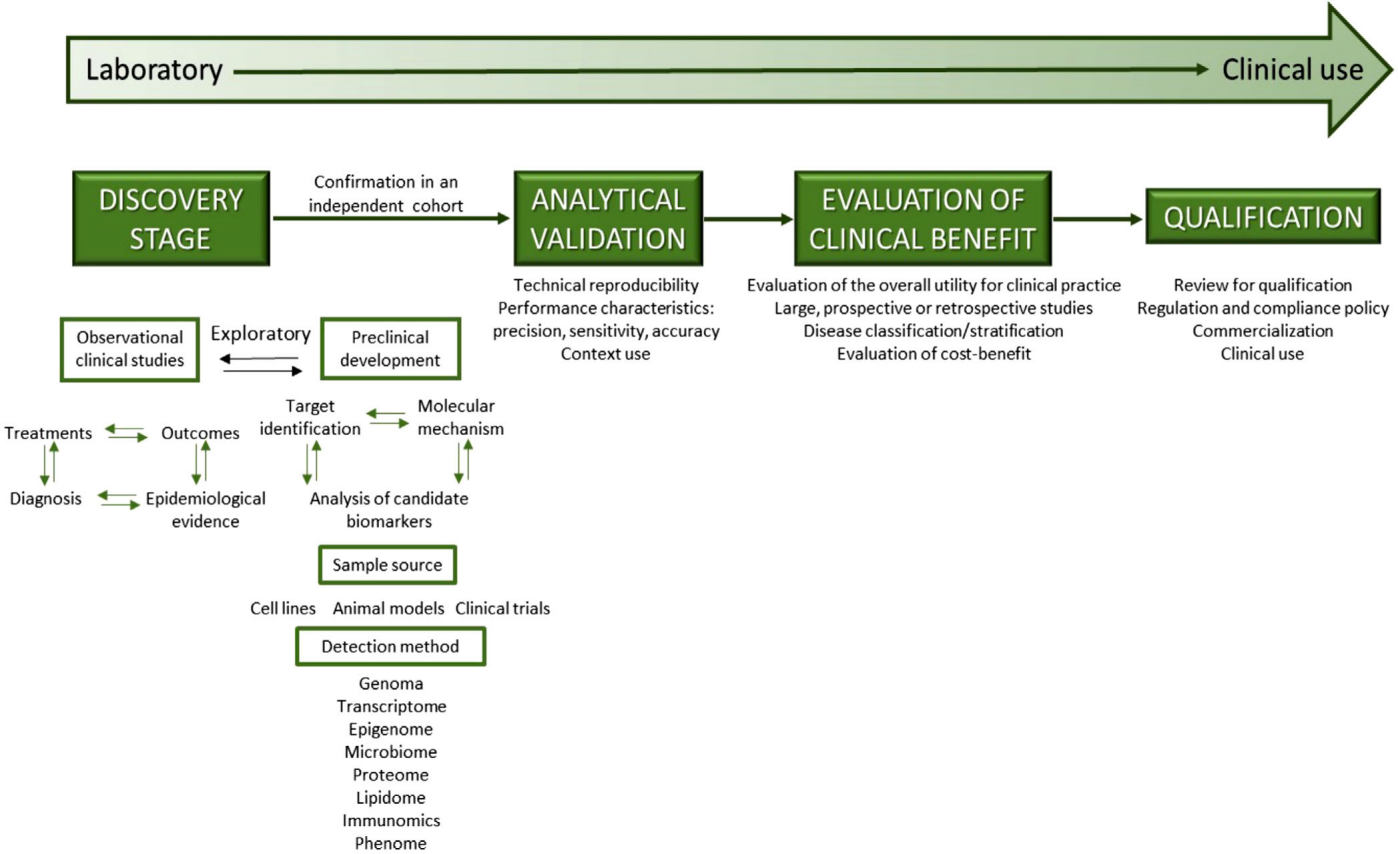
Biomarker development process from bench to bedside. Basic research in cancer biology provides preclinical data and new technology to support clinical advances in biomarkers discovery related to early detection (to determine specific healthy disorder), prognosis (to chart the likely course of the disease), prediction (to indicate drug response), and predisposition (to indicate risk of developing disease). Initially, clinical translational research had two main phases: (1) bench‐to‐bedside, in which new discoveries from the laboratory could be translated toward clinical research (proof of concept, phases I and II clinical trials) and (2) clinical use, in which these applications could be translated into the practice (phase III clinical trials, studies of clinical efficacy and development of guidelines). Recently, this process was further expanded by the addition of four more elements: discovery stage, analytical validation, evaluation of clinical benefits, and qualification

Unfortunately, HNC biomarkers studies are still hampered by several limitations due in part to the technical parameters such as sample size, suitable analysis strategy, standardized protocol for sample collection and storage, rational study design, detailed methods, as well as the complex nature of the disease itself.^5^ As noted above HNC is not a uniform disease, but a heterogeneous neoplasm with an array of genetic and epigenetic modifications associated with different risk factors.^5^ This certainly confers advantages for cancer cell division and survival, including growth factor‐independent proliferation, resistance to apoptosis, and an enhanced capability to overcome extracellular matrix (ECM) barriers and invade adjacent/distant tissues.^6^ Indeed, one of the most difficult barriers to overcome HNC invasion is the ability of cancer cells to disrupt the basal lamina, degrade ECM, and move beyond primary sites to gain access to lymphatic/blood vessels and establish metastasis. HNC is characterized by different invasive growth patterns, frequently associated with high risk of regional metastases and significant recurrences and secondary tumors.^7^

It is interesting to note that the majority of published studies in HNC focused on single signaling molecule/biomarker, often involving small number of patients with different tumor location, clinical stage, tumor grade, treatment, and no clear experimental design to address clinical questions. Besides, few investigations seem to shift their focus to multiplex gene/protein signatures, which can significantly improve diagnostic accuracy and enhance the predictive statistical power.^8^ The use of some biomarkers may become a reality in routine clinical management of HNC (Table 1), though the correlations with certain histopathological and clinical features are often inconsistently. Clearly, this is an open field and additional research is necessary to lend further insights into the molecular basis of HNC including large prospective well‐designed studies as well as deep mechanistic research are essential for validation and for the development of reliable indicators and tools for assessment of clinical events such as early diagnosis, recurrences, metastasis, and eventually culminating into better patient's outcome.

**TABLE 1:** Common gene/protein alterations and potential biomarkers in head and neck cancer

Cellular process	Gene	Protein	Type of gene	Significance/association
Cell cycle	*CDKN2A*	p16^INK4A^	Tumor suppressor	Decreased overall survival
	*TP53*	p53	Tumor suppressor	Decreased overall survival
	*CCND1*	G1–S‐specific cyclin D1	Oncogene	Nodal metastases; more rapid clinical course
	*CDKN1B*	p27	Tumor suppressor	Poor prognosis
	*MDM2*	Mdm2	Oncogene	Tumorigenesis
Growth signals	*EGFR*	EGFR	Oncogene	Nodal metastases; more rapid clinical course, consideration for targeted therapy
	*MYC*	c‐Myc	Oncogene	Tumor progression
	*RARB*	Retinoic acid receptor beta	Tumor suppressor	Decreased overall survival
Survival	*PIK3CA*	Catalytic p110α subunit of class 1 PI3Ks	Oncogene	High risk of recurrence
	*PTEN*	PTEN	Tumor suppressor	High risk of recurrence
WNT signaling	*FAT1*	Protocadherin FAT1	Tumor suppressor	Overall survival
	*AJUBA*	LIM domain‐containing protein AJUBA	Tumor suppressor	Treatment prediction
	*NOTCH1*	NOTCH1	Tumor suppressor	Chemosensitivity and overall survival
	STAT3	STAT3	Oncogene	Decreased overall survival

Source: http://www-ncbi-nlm-nih-gov.proxy3.library.mcgill.ca.

Moreover, immune system and tumor microenvironment (TME) are characterized by profound heterogeneity, dynamicity, and intercellular cross‐talks that add a new layer of complexity to define new paradigm in precision medicine (Figure 2).^9^ Investigation of the differences in HNC‐TME composition and their impact on cancer progression may help to understand the mechanism behind different tumor response, thus define possible targets for clinical intervention involving not a single modality treatment but rather a multitarget therapy. Recently, immunotherapy has provided promises and exciting treatment options for HNC, but the translation into clinical practice is limited to recurrent or metastatic cases. Pembrolizumab and nivolumab are the two PD‐1 antibodies approved by US‐FDA for the treatment of recurrent or platinum refractory metastatic HNC. However, no conclusions can be drawn on the role of PD‐L1 in identifying patients responding to immunotherapy, given that similar studies lead to contrasting results.^10^ A better understanding of the complex network between tumor, immune system, and combination of oncologic treatments will help us to refined patient selection and develop more efficient multimodality treatments.

**FIGURE 2:**
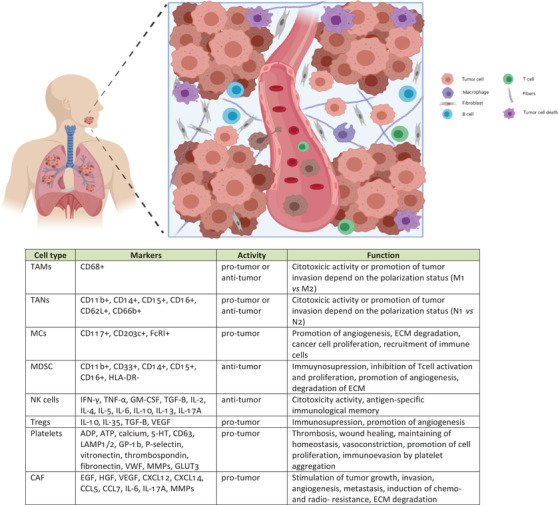
Different cell populations exhibit distinct functions within the tumor microenvironment (TME). In addition to the cancer cells, the tumor stroma is composed of many other supporting cell populations as well as the extracellular matrix, which crucially contribute to the tumor progression. The multiple different cell types include carcinoma‐associated fibroblasts (CAFs), endothelial cells, and inflammatory or immune cells (like neutrophils, macrophages, regulatory T cells, myeloid‐derived suppressor cells, natural killer cells, platelets, and mast cells). These cell subpopulations interact with each other and with the cancer cells via complex communication network through soluble factors (like growth factors, cytokines, chemokines, matrix proteins, and proteases) as well as proteins of the extracellular matrix (ECM). Abbreviations: TAM, tumor‐associated macrophage; TAN, tumor‐associated neutrophil; MCs, mast cells; MDSC, myeloid‐derived suppressor cell; NK, natural killer cell; Treg, regulatory T cell; CAF, cancer‐associated fibroblast; ECM, extracellular matrix (image created using Biorender Free Software)

In conclusion, we still do not fully understand HNC tumor behavior to manage this devastating disease. Given the deep biological heterogeneity and complex histological grade of this malignancy and its microenvironment, personalized therapeutic approaches possibly based on the use of combination of targeted therapy will provide a rational approach to improve treatments for patients with HNC. Understanding the aspects of the cell functionality and how molecular pathways interplay are the basis and the key strategy to develop new therapeutic approaches to improve patient's prognosis.

## FUNDING INFORMATION

Global Affair/DFATD (#249584), Brazil‐Canada (#249569), RSBO#80596; NCOHR (New Frontier Seed Grant); FAPESP.

## CONFLICT OF INTEREST

The authors declare that there is conflict of interest.
